# *Ipomoea aquatica* Extract Shows Protective Action Against Thioacetamide-Induced Hepatotoxicity

**DOI:** 10.3390/molecules17056146

**Published:** 2012-05-22

**Authors:** Salim Said Alkiyumi, Mahmood Ameen Abdullah, Ahmed Salim Alrashdi, Suzy Munir Salama, Siddig Ibrahim Abdelwahab, A. Hamid A. Hadi

**Affiliations:** 1 Department of Molecular Medicine, University of Malaya, 50603 Kuala Lumpur, Malaysia; 2 Department of Pharmacy, University of Malaya, 50603 Kuala Lumpur, Malaysia; 3 Department of Chemistry, University of Malaya, 50603 Kuala Lumpur, Malaysia

**Keywords:** *Ipomoea aquatica*, hepatoprotective, thioacetamide, oxidative stress

## Abstract

In the Indian system of traditional medicine (Ayurveda) it is recommended to consume *Ipomoea aquatica* to mitigate disorders like jaundice. In this study, the protective effects of ethanol extract of *I. aquatica* against liver damage were evaluated in thioacetamide (TAA)-induced chronic hepatotoxicity in rats. There was no sign of toxicity in the acute toxicity study, in which Sprague-Dawley (SD) rats were orally fed with *I. aquatica* (250 and 500 mg/kg) for two months along with administration of TAA (i.p injection 200 mg/kg three times a week for two months). The results showed that the treatment of *I. aquatica* significantly lowered the TAA-induced serum levels of hepatic enzyme markers (ALP, ALT, AST, protein, albumin, bilirubin and prothrombin time). The hepatic content of activities and expressions SOD and CAT that were reduced by TAA were brought back to control levels by the plant extract supplement. Meanwhile, the rise in MDA level in the TAA receiving groups also were significantly reduced by *I. aquatica* treatment. Histopathology of hepatic tissues by H&E and Masson trichrome stains displayed that *I. aquatica* has reduced the incidence of liver lesions, including hepatic cells cloudy swelling, infiltration, hepatic necrosis, and fibrous connective tissue proliferation induced by TAA in rats. Therefore, the results of this study show that the protective effect of *I. aquatica* in TAA-induced liver damage might be contributed to its modulation on detoxification enzymes and its antioxidant and free radical scavenger effects. Moreover, it confirms a scientific basis for the traditional use of *I. aquatica* for the treatment of liver disorders.

## 1. Introduction

*Ipomoea aquatica*, which belongs to the family Convolvulaceae is supposed to be originated in China and is usually consumed as a green leafy vegetable [1]. This plant is widely distributed all around the World, especially India, Malaysia, Indonesia, China, Hong Kong and some parts of USA [2]. This plant is grown as an aquatic plant which grows abundantly in marshy areas. Various parts of the *I. aquatica* plant are used medicinally in Southeastern Asia and reported to be useful for the treatment of high blood pressure [3], as an emetic in the treatment of opium and arsenic poisoning [4]. The dried juice has reported to be a purgative [4], while the leaves and stems possess cooling action. Moreover, it is also traditionally used in the treatment of nervous and general debility, piles, worm infections, leucoderma, leprosy, jaundice and liver complaints [2]. Very few studies have been done in this plant. This includes the inhibition of prostaglandin synthesis [5], eye diseases, constipation [6] and hypoglycaemic effects [7]. Phytochemical investigations of this plant have revealed the presence of carotenes such as β-carotene, cryptoxanthin, lutein, lutein epoxide, violoxanthin and neoxanthin [8], flavonoids such as mycertin, quercetin, luteolin and apigenin [9] and some alkaloids [10]. 

Even though the liver is the most significant organ of our body for detoxification, disorders to this organ remain some of the most serious health problems [11]. Liver diseases such as hepatocellular carcinoma, viral and alcoholic hepatitis and non-alcoholic steatosis are the most common liver-associated and prevalent diseases in the World, which are closely associated with jaundice [12]. As modern medicine fails to cure these ailments completely and harmlessly, research on natural products has gained much attention in the drug discovery against diseases such as liver diseases [13]. Most of the time the research will be guided by some traditional practice or from undocumented treatment modalities [14,15]. In the Indian system of traditional medicine (Ayurveda) it is recommended to consume *I. aquatica* to mitigate disorders like jaundice [2]. The effectiveness of these plant products from traditional claims must be proved to help develop noval drugs acting against these disorders. Nevertheless, no literature was found on the hepatoprotective action of this plant. Keeping this in view, the present study was aimed to evaluate the hepatoprotective activity of *I. aquatica* on thioacetamide-induced liver damage in rats. 

## 2. Results and Discussion

### 2.1. Biochemical Parameters of the Liver

In acute toxicity studies, the extract was found to be safe up to 5 g/kg. No mortality or toxic symptoms were observed during the entire duration of the study. Many types of chemical agents have been employed previously for the indication of hepatic damage. Thioacetamide (TAA), which has been used in the present study, is a well-established tool to induce hepatotoxicity in experimental animals models [16,17]. TAA is able to produce different grades of hepatic damage, including centrilobular necrosis [18], inflammation and suppression of anti-oxidant activity [19]. In the current study the hepatic damage occurred was assessed by measuring serum levels of specific liver enzymes such as ALP, ALT, AST and bilirubin. Even though these enzymes were found to be elevated in the TAA treated group of animals due to the leakage of the enzymes from liver as a result of tissue damage, the plant extract treatment was found to significantly (*p* < 0.001) restore the enzyme levels both at 250 and 500 mg/kg. These results were well compared with the standard drug, silymarin (Table 1).

**Table 1:** The effects of *I. aquatica* extracts and silymarin on the biochemical parameters in serum of TAA intoxicated rats.

Group	ALP IU/L	ALT IU/L	AST IU/L	Protein g/L	Albumin g/L	Bilirubin g/L	Prothrombin time (PT)
Control (N.S)	76.23 ± 0.47	37.8 ± 0.23	60.9 ± 0.75	68.65 ± 0.44	25.8 ± 0.32	1.23 ± 0.06	0.85 ± 0.01
TAA+N.S	234.1 ± 0.28 *	160.8 ± 0.4 *	221.63 ± 1.45 *	48.88 ± 0.33 *	8.68 ± 0.34 *	4.73 ± 0.40 *	1.67 ± 0.03 *
TAA+ Silymarin	80.36 ± 0.65 **	38.13 ± 0.35 **	66.01 ± 0.27 **	66.81 ± 0.54 **	24.76 ± 0.53 **	1.24 ± 0.03 **	0.91 ± 0.01 **
TAA+ *I. aquatica* 250mg/kg	87.76 ± 0.43 **	83.83 ± 0.25 **	89.33 ± 0.57 **	63.03 ± 0.63 **	19.68 ± 0.34 **	2.42 ± 0.09 **	1.13 ± 0.02 **
TAA+ *I. aquatica* 500 mg/kg	77.8 ± 0.46 **	39.85 ± 0.33 **	63.73 ± 0.30 **	67.61 ± 0.27 **	24.93 ± 0.45 **	1.25 ± 0.03 **	0.90 ± 0.02 **

Data are expressed as mean ± SEM. Means among groups (n = 6 rats/group) show significant difference, * p < 0.001 vs. normal group, ** p < 0.001 vs. cirrhosis group.

In most instances, the serum bilirubin levels must be elevated in hepatic jaundice [20]. We have observed a similar trend in the elevation of serum billurubin level during TAA traement, and concurrent reduction with the plant extract. It is well known that following cellular damage the capacity to synthesize proteins is reduced, and as the extent of damage increases, the levels of these proteins in the plasma will tend to decrease [21]. A similar observation was seen in our studies, where the total protein and total albumin levels in the serum were markedly decreased, but as seen in the silymarin treated group, the plant extract also restored these levels of protein to normal signifying a return from the abnormal state to normal. 

### 2.2. SOD, CAT and MDA Contents in Liver Homogenates

Biological systems have protective arrangements which defend them against the harmful effects of free radicals. This includes SOD and CAT [22]. In the liver tissues of the hepatotoxicity control group, the antioxidant activity of SOD and CAT were significantly (*p* < 0.001) reduced as compared with the normal animal group. In hepatotoxicity these enzymes are structurally and functionally weakened by the radicals, resulting in liver damage [23]. Meanwhile, the treatment group with both low and high dose of plant extract succeed in reversing the enzyme levels back by protecting the tissues from attack by free radicals. 

Due to the involvement of several pathological conditions, recently much attention has been paid to free radical peroxidation (Table 2). Lipid peroxidation, which refers to the oxidative degradation of lipids, is a multifarious and natural harmful process [24]. MDA level increases in the tissue represent an indirect index of lipid peroxidation. The increase in liver TBARS indicates enhanced lipid peroxidation leading to tissue injury and failure of the anti-oxidant defense mechanisms to prevent the formation of excess free radicals [11]. In the present study, thioacetamide induced a rise in MDA level has was significantly (*p* < 0.001) reduced by the extract (Table 2).

**Table 2:** SOD, CAT and MDA contents in liver homogenates after two months treatment.

Group	SOD	CAT	MDA
	U/mg protein	U/mg protein	U/mg protein
**Control (N.S)**	18.03 ± 0.24	38.76 ± 0.32	1.19 ± 0.04
**TAA+N.S.**	8.63 ± 0.32 *	18.73 ± 0.32 *	4.80 ± 0.06 *
**TAA+ Silymarin**	14.68 ± 0.21 **	37.13 ± 0.40 **	1.7 ± 0.04 **
**TAA+*I. aquatica* 250 mg/kg **	12.41 ± 0.27 **	26.46 ± 0.64 **	2.33 ± 0.09 **
**TAA+*I. aquatica* 500 mg/kg **	14.56 ± 0.29 **	37.95 ± 0.24 **	1.40 ± 0.09 **

Data are expressed as mean ± SEM. Means among groups (n = 6 rats/group) show significant difference, * p < 0.001 vs. normal group, ** p < 0.001 vs. cirrhosis group, *** *p < 0.05 vs. *cirrhosis group.

### 2.3. Histopathological Study of the Liver

**Figure 1:**
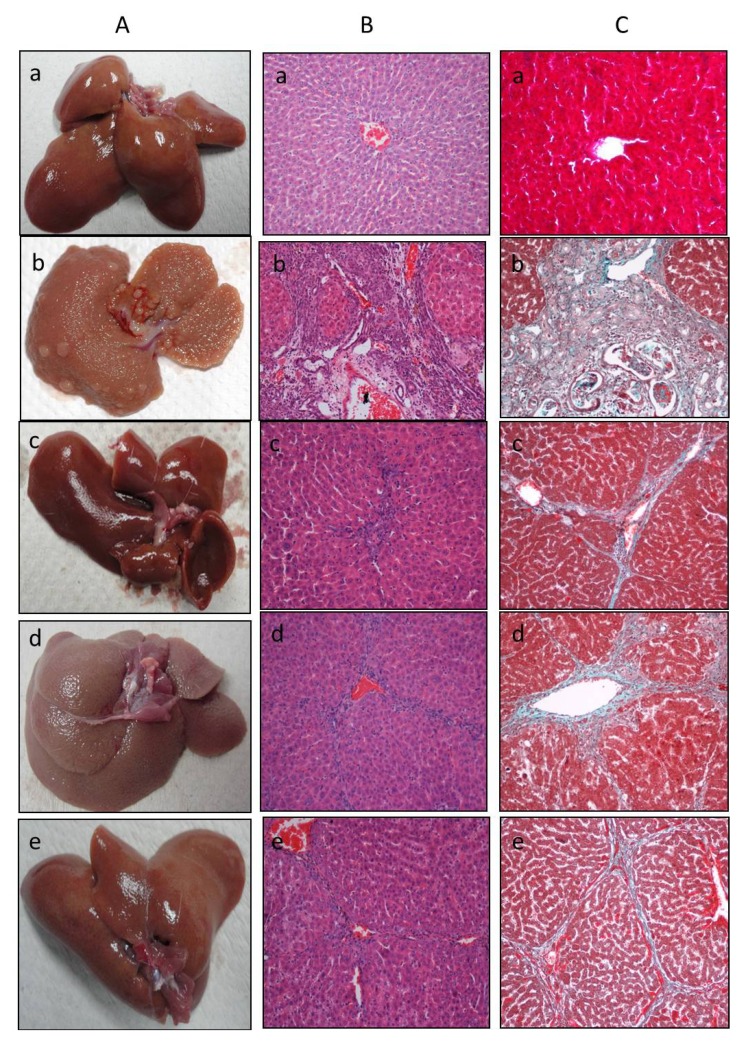
Effects of *I. aquatica* extract on thioacetamide induced liver damage in rats. (a) Control group, (b) animals treated with thioacetamide, (c) animals treated with thioacetamide and silymarin, (d) animals treated with thioacetamide and *I. aquatica* 250 mg/kg and (e) animals treated with thioacetamide and *I. aquatica* 500 mg/kg. (**A**) Gross morphology, (**B**) Hematoxylin/eosin stain, (**C**) Masson trichrome stain; magnification 20×.

The overall liver morphological changes and fibrosis induced by TAA administration were demonstrated by qualitative histopathological examination. The normal control animals showed regular hepatocytes with well-preserved cytoplasm, prominent nucleus, nucleolus and central vein in the histological profile. There was no sign of inflammation, fatty change and necrosis in these animals (Figure 1A,a and Figure 1B,a). 

In animals administered with thioacetamide alone, liver sections showed marked congested central veins, sinusoid and multifocal area of necrosis, fatty changes and inflammatory cell with granular swelling (Figure 1B). In addition, the MT stain clearly demonstrated that fiber extension and collagen accumulation (Figure 1C,b) was also present. A high degree of vacuolation followed by marked congested veins, multifocal area of necrosis, fatty changes and inflammatory cells are substantial structures in the histology of a damaged liver [25]. Whereas the histological architecture of liver sections of the rats treated with the plant extract showed more or less normal lobular pattern with a mild degree of fatty change, necrosis and lymphocyte infiltration almost comparable to the control and silymarin treated groups. The plant extract exposure greatly improved liver morphological changes, fibrosis and necrosis, which is clearly seen in both Figure 1ABC,e. and well compared with control and Silymarin treated group.

## 3. Experimental

### 3.1. Plant Material

The plant material of *I. aquatica* was purchased from the Ethno Sdn Bhd. The plant was identified, and voucher specimen was kept in our laboratory for future reference. The plant material was cut into small pieces, which were first washed with tap water and then with distilled water. The plant was then air dried for 7 days, after which the dried leaves were ground into a fine powder using a grinder.

### 3.2. Extraction Procedure

The dried plant was powdered (approximately 100 g) and was extracted with 95% ethanol (900 mL) for 72 hours at room temperature. The suspension was stirred from time to time to allow the powder to fully dissolve in the ethanol. The ethanol extract was then filtered through cheesecloth, followed by filter paper (Whatman No. 1) and evaporated under low pressure by using Buchi-type rotary evaporator to a percentage yield of crude-dried extract of 18.4% (w/w).

### 3.3. Experimental Animals

The study was carried out using male Sprague-Dawley rats (195–215 g) obtained from the Animal House Unit, Faculty of Medicine, University of Malaya, Malaysia. The chosen animals were housed in bottomed cages at 25 ± 3 °C temperature, 50–60% humidity, and under a 12  h light-dark cycle for a week before the experiment. The animals were maintained at standard housing conditions and free access to standard diet and water *ad libitum* during the experiment. The experiment was performed in accordance to the guideline of taking care of animals prepared by the National Academy of Sciences. 

### 3.4. Chemicals

All chemicals used in the experiment were of analytical grade and purchased from Sigma-Aldrich. Thioacetamide (Sigma-Aldrich, St. Gallen, Switzerland) was used as an inducer of liver cirrhosis and was dissolved in sterile distilled water and injected intraperitoneally to the experimental rats in concentrations of 200 mg/kg body weight [26]. Silymarin (International Laboratory, South San Francisco, CA, USA) used as a standard drug in the experiment, was dissolved in distilled water and orally administered to rats in dosage of 50 mg/kg body weight [27].

### 3.5. Acute Toxicity Test

The procedure for this animal study was reviewed and then approved by Animal Ethics Committee (Ethic No. PM/27/07/2010/MAA). The acute toxicity study was undertaken employing a total of 36 Sprague-Dawley rats. Eighteen male and 18 female rats ranging between 7–8 weeks of age procured from the Animal House Unit, Faculty of Medicine, University of Malaya, Malaysia. The animals were divided into three groups with six rats each marked as vehicle (distilled water, 5 mL/Kg, 2 g/kg and 5 g/kg) of prepared extract, respectively. Prior to dosing the rats were fasted overnight and after the dose for another four hours. Following the dose the rats were carefully watched for the development of any clinical or toxicological symptoms at 30 min and then 2, 4, 8, 24 and 48 h. On the 15th day all the rats were sacrificed and samples for biochemical and histological studies were collected and analyzed per standard methods [28].

### 3.6. Preparation of Doses

Distilled water was used to dissolve the crude-dried ethanol extract before oral administration to experimental rats. Plain distilled water was used to dissolve the standard drug, silymarin (50 mg/kg). The doses were prepared fresh each day. 

### 3.7. Protocol for Hepatoprotective Study

Thirty rats were randomly divided into five groups of six animals each. Group 1 (normal control) received intraperitoneal injection of sterile distilled water (1 mL/kg) three times a week for two months and oral administration of distilled water (5 mL/kg) for two months. Group 2 (positive control) received intraperitoneal injection of TAA (200 mg/kg) three times a week for two months and oral administration of distilled water (5 mL/kg) for two months. Group 3 (standard drug control) received intraperitoneal injection of TAA (200 mg/kg) three times a week for two months and oral administration of Silymarin (50 mg/kg) for two months. Group 4 (low dose ethanol extract treated group) received intraperitoneal injection of TAA (200 mg/kg) three times a week for two months and oral administration of *I. aquatica* (250 mg/kg) for two months. Group 5 (high dose ethanol extract treated group) received intraperitoneal injection of TAA (200 mg/kg) three times a week for two months and oral administration of *I. aquatica* (500 mg/kg) for two months.

### 3.8. Assessment of Liver Function

Biochemical parameters were analyzed by a standard biochemistry automated analyzer at the Central Diagnostic Laboratory of the Medical Centre of University Malaya. Collected blood samples from the rats were centrifuged at 3,000 rpm for 10 min. Then the separated serum samples were used to analyze liver biochemical parameters, including alanine aminotransferase (ALT), aspartate aminotransferase (AST), alkaline phosphatase (AP), total protein, bilirubin and albumin. 

The liver samplez were rinsed with saline and then homogenized (10% w/v) in 50 mM cold potassium phosphate buffer (pH 7.4), and thereafter centrifuged at 3,500 rpm for 10 min at 4 °C. Then the supernatant was separated and saved for malondialdehyde level (MDA) analysis as per the thiobarbituric acid procedure [29,30], which is an indicator for lipid peroxidation levels. Superoxide dismutase (SOD) and catalase (CAT) were analyzed by Cayman’s SOD and catalase assay kits.

### 3.9. Histopathological Examination

Liver histopathological examinations were carried out by means of a light microscope. Liver was fixed in 10% buffered formaldehyde, and thereafter processed by programmed tissue processing machine and after that embedded in paraffin wax. Following embedding a 5 μm thick section was prepared and stained with hematoxylin-eosin (HE) and Masson-Trichrome (MT) for photomicroscopic assessment [31]. 

### 3.10. Statistical Analysis

All of the values are reported as mean ± S.E.M. The statistical significance of variations between groups was analyzed employing one-way ANOVA pursued by Tukey Post-Hoc test analysis using SPSS version 18 (SPSS Inc. Chicago, IL, USA) with a value of *p* < 0.05 was regarded significant when compared to the control group.

## 4. Conclusions

In conclusion, the results of this study demonstrate that the *I. aquatica* was effective in prevention of TAA-induced hepatic damage in rats. Our results show that the protective effect of *I. aquatica* in TAA-induced liver damage might contribute to its modulation on detoxification enzymes and its antioxidant and free radical scavenger effects. Moreover, it confirms use of *I. aquatica* traditionally for the treatment of liver disorders.
